# Hospital Organizational Structure and Information Processing: An Entropy Perspective

**DOI:** 10.3390/e25030420

**Published:** 2023-02-26

**Authors:** Windi Winasti, Hubert Berden, Frits van Merode

**Affiliations:** 1 IQ Healthcare, Radboudumc, 6525 EP Nijmegen, The Netherlands; 2 Elisabeth-TweeSteden Ziekenhuis, 5022 GC Tilburg, The Netherlands; 3 Care and Public Health Research Institute, Maastricht University, 6200 MD Maastricht, The Netherlands; 4 Maastricht University Medical Centre+, 6229 HX Maastricht, The Netherlands

**Keywords:** hospitals, information processing, coordination, nurses, entropy

## Abstract

Organizational structure enables organizations to achieve their goals. The chosen organizational structure determines, to a large extent, the flow of information streams and the manner and extent to which roles, power, and responsibilities are delegated and coordinated to achieve the organization’s goals. In this study, we applied information theory with entropy as the central concept to assess the effectiveness and costs of an organizational structure and its coordination processes. Entropy was used to measure the amount of uncertainty associated with probabilistic events. In the context of organizational design, entropy values can be assigned to specific organizational structures to gain insights into the factors that lead to delays in decision-making. We used Shannon’s entropy theory to quantify Galbraith’s organizational structure and coordination process as applied to the perinatology care system of Radboud University Medical Centre in the Netherlands. Our entropy analysis provided insights into how departments should be partitioned and which coordination mechanisms should be used to achieve organizational goals, such as minimizing delays in decision-making. Particularly, two types of entropy appear to be important: positional entropy and task allocation entropy. These are different dependent variables on the organizational design scenarios. Our analysis shows that entropy is one method to determine optimal organizational structures and coordination processes. Entropy can be used as a concrete way of assessing the effectiveness of organizational design given the level of uncertainty of the environment and the required speed of decision-making.

## 1. Introduction

Organizations such as hospitals exist to achieve goals. Hospitals aim to ensure that patients receive the proper care at the right time, in the right place, from the right caregivers. The organizational structure allows organizations to achieve their goals. The chosen organizational structure determines, to a large extent, the manner and extent to which roles, power, and responsibilities are delegated and coordinated to achieve the organization’s goals. The structure also provides, to a large extent, insights into what and where information streams should flow in the organization. As certain organizational structures follow certain information processing structures [1,2], a hospital organization can be considered an information processing organization. Following Galbraith, coordination problems can often be identified as information processing problems.

**Figure 1:**
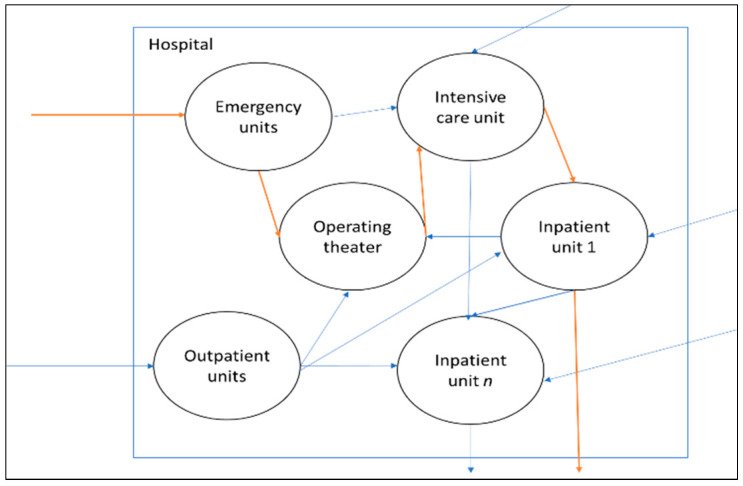
Patient care is delivered through a network of functionally independent departments (e.g., emergency department (ED), outpatient units (OUs), intensive care unit, and inpatient wards (IWs)) within a hospital system. In general, acute patients arrive via the ED, while elective patients arrive via OUs. Subsequent phases of the patient care trajectory connect various capacity resources such as IWs, surgical procedures in operating theaters (OTs), and intensive care unit. Different patient care trajectories are connected through shared resources.

An example of patient movement (flow) in a hospital is shown in Figure 1. A hospital system with various independent departments can be characterized as a complex queuing system [3]. Previous studies showed that uncontrollable queues develop in such a complex system due to the systemic problems arising from the hospital structure [3,4]. Systemic failures appear when local blockages result in further sequences of local blockages. For instance, a specific patient flow increase could lead to blockages in another patient’s care trajectory, which seems at first sight unconnected to the blocked shared resource. Blockage problems require hospitals to have feedback mechanisms in order to adjust their input capacity to meet the target output [5].

Referring to the example shown in Figure 1, given the structure of the hospital organization, information is needed to coordinate the flow of patients to other departments in the hospital system. One important piece of information is positional information [6], which refers to a patient’s actual position in the system. Although positional information is essential, hospitals still need to check the patient’s detailed information status (e.g., obtaining better or remaining ill) to see whether the patient is ready to leave the hospital (i.e., recovered). Knowing only the physical location of a patient is often insufficient to ensure whether the planned capacity of the hospital needs adjustment or not, making hospital operations more complex than manufacturing operations. The design solution for this would be to increase the frequency of information checks so that the decisions on whether or not the planned capacity needs adjustments to remain accurate. For instance, efficient patient flow in the intensive care unit may involve assessing patients currently in the intensive care unit every two hours to determine whether they can be discharged to an inpatient unit.

Such assessments cost information processing time. The more assessments, the more time is lost, which can increase information processing costs. Furthermore, the information must be shared among departments to coordinate patients’ movements. Sharing and coordinating such information also costs time, while processing too much information, as well as a lack of information, can require extensive time, leading to a delay in decision-making. Hence, efficiency in acquiring and processing feedback from other interdependent departments is crucial in minimizing delays in decision-making. Finding the right balance between the frequency of feedback control (i.e., when to process and coordinate information) and the cost is becoming an important research topic for organizations [7].

### 1.1. Entropy and Information Processing Organizations

An information entropy model can measure the amount of information associated with specific (organizational) structures [6,7,8]. Entropy is defined as a measure of randomness or disorder in a system [9,10]. Entropy measures the amount of uncertainty associated with probabilistic events [8]. According to Shannon [8], the higher the uncertainty in the system, the higher the entropy; consequently, more information is required to understand what is happening in it. The entropy concept has been applied in various fields, such as statistical thermodynamics, economics, operations research, queueing theory, manufacturing, and many more [6]. In an information processing organization, entropy can measure the uncertainty related to the work that has to be performed in order to obtain information about specific events. For instance, information entropy values can be assigned to specific organizational structures to gain insights into the factors that lead to effective decision-making.

### 1.2. Contributions of This Study

The literature suggests that organizations need to build information processing capability to minimize decision-making delays in organizations [6,11,12]. Timely information processing depends on the effort made to gather and share information with others [6,7]. The organizational information theory has been used extensively in investigating manufacturing operations [11,13], but only a few times in hospital operations [12,14]. Hospital patient flow problems can be minimized as delays in processing and coordinating information are also minimized [7]. Moreover, although recent studies have shown relevant results from evaluations of hospital organizational structures [15,16], to the best of the authors’ knowledge, none precisely followed Galbraith’s [1] integrated design strategies in a hospital, using entropy as the method to quantify each strategy. Following that, as suggested by Weick [17], the distribution of authority within the organization must also change when the organizational structure changes. A recent review of various hospital coordination mechanisms is given by Tippong et al. [18]. Our study contributed to the current understanding of how an entropy-based criterion can be used to assess the effectiveness and costs of the organizational structure and its coordination processes for hospital operations. In concrete terms, we answer the following questions:

- What is the optimal care system configuration (i.e., how departments are partitioned) to minimize uncertainty?
- How does the distribution of authority minimize uncertainty?
- How can the entropy concept be deployed to evaluate hospital organization and information processing?

## 2. Information Processing and Delays in Decision-Making

In this section, we present the theory behind the application of information processing theory for minimizing decision-making delays in organizations. In Section 2.1, we explain how hospital organizations can use a closed-loop system to balance differentiation and integration to adapt to environmental changes. We then present Galbraith’s [1] four integration design strategies in Section 2.2 for hospitals to enable the closed-loop mechanism. Finally, in Section 2.3, we present two types of structural entropies that can be used to measure the four integration design strategies.

### 2.1. Integration versus Differentiation

According to a study by Lawrence and Lorsch [19], an organization is defined as “a system of interrelated behaviors of people who are performing a task that has been differentiated into several distinct subsystems, each subsystem performing a portion of the task, and the efforts of each being integrated to achieve the effective performance of the system”. As Lawrence and Lorsch [19] argued, organizations must balance differentiation and integration to succeed. In their study, companies that managed to achieve high subsystem differentiation while maintaining high integration between subsystems seemed to be best equipped to adapt to environmental changes. Lawrence and Lorsch [19] explained how as an organization becomes differentiated into subsystems, its environment becomes segmented in order to undertake whole tasks (with their uncertainty) in the related environment. Hence, the kind of integration needed to achieve effective performance depends on the characteristics of the tasks in the organization’s segments.

As an organization segments its environment, the accompanying uncertainty can disturb the organization in various ways. Hence, as Weick [17] suggested, the organization needs multiple options to solve it. According to Weick’s [17] model of organizing, situations like this are best resolved through collective interdependent communication and information processing activities that help reduce uncertainty and identify appropriate responses. One option to deal with uncertainty is to manage the distribution of authority within the organization. For instance, if a particular segment of the organization has a task environment that is not shared with other segments, and this segment also does not share resources with other segments, the kind of integration, such as central coordination of all decisions of this particular segment, would be costly and highly inefficient. Instead, the design solution is to transfer the decision points downward in the hierarchy to the organizational segment where the action takes place [20].

Another way to deal with uncertainty is to manage a hospital (and the decentralized departments) as a closed-loop system [5,21] With the closed-loop system, hospitals maintain high integration between departments, although they, on the other hand, may have high department differentiation. This is because a closed-loop system is designed so that feedback is signaled to the input to achieve and maintain the desired output condition [22]. To avoid blockages, decisions to match the supply demand of departments are based on feedback from each department and other interrelated departments. To enable decision-making in a closed-loop system, hospitals can create mechanisms that permit coordinated action across many interdependent departments [5].

### 2.2. Information Processing Organizations in Hospitals

To enable decision-making in a closed-loop system, certain coordination mechanisms are required. Galbraith [1] argued that having rules, providing programs, and setting goals are more straightforward approaches for an organization’s coordination mechanisms. Staff members working on different subtasks are taught about possible situations and events that could occur while performing each subtask [1]. However, such a coordination mechanism may be a good solution for small companies, but it is only feasible in some organizations. In the case of an unforeseen event, management becomes a challenge, though this is solvable by hierarchy [1]. We refer to this as “management by exception”, as Galbraith [1] explained. In the case of an increasing number of unforeseen events, a hierarchy can reach its maximum capacity for processing information, resulting in an overload.

Galbraith [1] then explained four integration design strategies for information processing organizations, in addition to the rules, programs, goals, and hierarchy mentioned above. The choice of a specific strategy depends on its relative cost and the uncertainty the organization faces. Hence, organizations may differ in their structure, though they can be equally effective.

**Figure 2:**
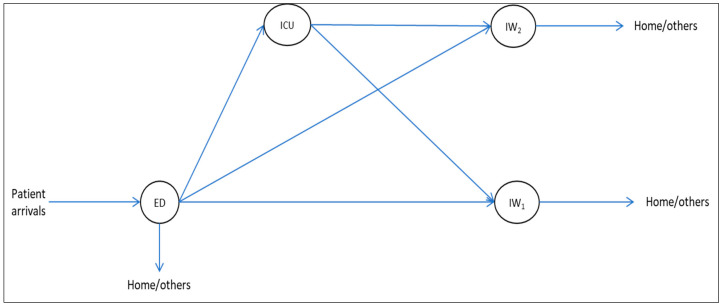
Hospital system with M departments.

To illustrate Galbraith’s [1] design strategy in a hospital system, we converted the example in Figure 1 into a mini-hospital with only four departments, as shown in Figure 2. The example departments are the emergency department (ED), where patients arrive, an intensive care unit (ICU), and two surgical/medical wards (IWs). The decision authority regarding patient movements is coordinated in a decentralized manner, which means the involved departments coordinate patients separately from the other departments. Here, the departments need the patients’ positional information to know their actual position in the system [6]. The positional information can be acquired through the organization’s information system. As stated earlier, although positional information is essential, hospitals still need to check the detailed status of patients (e.g., obtaining better or remaining ill) to see whether they are ready to leave the hospital (i.e., recovered). The acquisition of such fine-grained information can contribute to delays in decision-making.

Furthermore, due to the division of labor in a hospital, nurses are allocated to departments in a decentralized manner by a local planner in each department. Here, the local planner needs task allocation information, often in fine granularity, to determine which types of nurses to deploy to which department beds for a specific shift.

Uncertainty about the rate of patient arrivals, the length of stay, or the care pathways in a hospital necessitates hospitals to acquire and process information to make planning adjustments. The first two designs of Galbraith’s [1], the slack resources and the creation of self-contained tasks, aim to minimize the need for information processing. The other two designs increase the capability to process information for decision-making. These four strategies are related to the central point of Lawrence and Lorsch [19], where the choice of an integration strategy depends on the characteristics of the tasks (uncertainty) in the organization’s segments.

- Slack resources

The creation of slack resources is about creating buffers to reduce the effect of uncertainty on organizational segments. Creating slack resources may be necessary to accommodate disruptions or peaks in demand [23,24]. The design choices for creating buffer resources comprise the what (e.g., lead time, utilization, etc.), the when and the where in the organization.

In the example given in Figure 2, the hospital can create spare beds (i.e., a buffer) in the inpatient wards (IW1 and IW2). Creating a buffer in the inpatient wards reduces the need for information processing when more acute patients arrive from the ED and the ICU than anticipated. This is because a mismatch between the number of inpatient beds and the patient demand for inpatient beds would be less likely to occur. Although uncertainty always requires some buffering, the required number and size of buffers can be mitigated with capacity pooling [23,25,26].

When inpatient wards are physically pooled, from the perspective of decision-makers, the complexity of patient allocation to wards is reduced as the number of states to be considered decreases. In this case, a portion of patient positional information processing belonging to the pooled wards is now delegated to the pooled wards. Reduced complexity from the system perspective can lead to minimized delays in decision-making.

**Figure 3:**
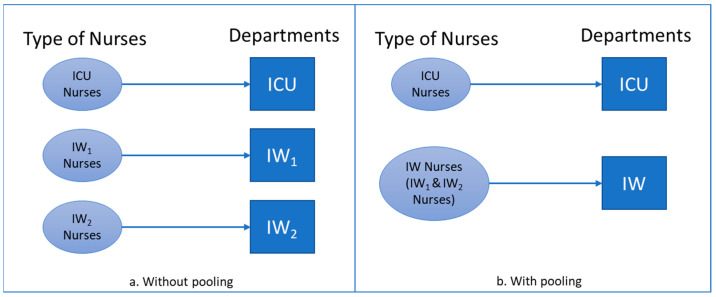
(**a**) Without and (**b**) with physical pooling of inpatient wards.

Furthermore, the nurses are now multi-skilled within the pooled wards and can be assigned to them, as can be seen in Figure 3b. When the planner (i.e., the one who makes the decision to allocate nurses to beds in the pooled wards) is centralized along with the nurses, as the wards are physically pooled, the complexity of nurse allocation to wards from the hospital system perspective is also reduced, as the number of states to be considered (i.e., types of nurses and wards) decreases. The system then needs less information to complete the work.

- Self-contained task

In a self-contained design, the functional grouping of resources is carried out based on the product category [1]. This design minimizes the need for information processing because it reduces the amount of output diversity (and hence its uncertainty) that would occur with a single collection of resources.

For the hospital example shown in Figure 2, resource departments are grouped according to the nature of the illness (e.g., neurological or orthopedic inpatient wards) and the type of bed (e.g., intensive care or regular beds in inpatient wards). Once diagnosed in the ED, a patient can progress to the neurological ward, the orthopedic ward, or the intensive care unit. Supposing the hospital focuses its grouping only on the type of bed, the output diversity is reduced to only intensive care or regular beds in wards (i.e., there is no distinction between neurological and orthopedic inpatient wards). This design eventually reduces the division of labor (i.e., from three groups to two groups), and thus the needed information processing to allocate patients to wards. However, the coordinated effort in the regular wards increases as the number of choices (i.e., multi-skilled nurses) increases.

A coordinated effort for nurse allocation in the regular wards requires less information to complete the work when the number of states to be considered (i.e., types of nurses and wards) decreases.

- Investment in vertical information system

Given the uncertainty (e.g., patient arrivals), exceptions such as mismatches between supply (e.g., of beds) and demand (e.g., for beds) require hospitals to adjust their plan accordingly. Galbraith [1] explained “management by exception,” which occurs when the subordinate reports the mismatches to the manager when the supply–demand mismatch is outside of the subordinate’s scope. The subordinate waits for the manager’s instructions to solve the unforeseen mismatch. In the case of increasing mismatches, a hierarchy can reach its maximum capacity for processing information, resulting in an overload. Investing in a vertical information system is then needed to enable feedback from the operational level to cascade up to the tactical level, where there is a global overview of all affected subsystems, without overloading the hierarchical communication channels.

As discussed in Galbraith [1], the greater the frequency of re-planning, the greater the amount of resources required to process information to fulfill the adjustments. Galbraith [1] suggested that the cost of information processing can be minimized if the language is formalized. Formalizing the decision-making language means that “more information is transmitted with the same number of the symbol”. Formalization can also be achieved through information aggregation, in which the granularity of information is reduced. This involves the concept of coarse graining. A fine-grained description is a detailed description of the system’s microscopic behavior, while a coarse-grained description is one where the fine detail has been smoothed over through aggregation.

Several methods of coarse graining can be used. Gong et al. [6] discussed how a manufacturing system worked with aggregated information to plan and control its production using a CONWIP system. In CONWIP, the system authorizes the release of patients based on system status [23,27]. The system status here is information aggregated by workload.

For the hospital example shown in Figure 2, feedback from the ICU and IWs is needed to know if patients can be admitted from the ED accordingly. When the information from all of the wards is aggregated only to report their workload (i.e., the number of occupied beds), instead of every detail on patient mismatches, the hospital can have a shorter planning horizon (i.e., greater feedback frequency) to acquire and process information from interdependent wards. Due to the aggregated information (i.e., workload as the number of occupied beds), this design eventually reduces the output diversity, hence the need for information processing to allocate patients to wards.

- Creation of lateral relationships

In a hospital, patients move through different departments to obtain their needed treatments, and in this way, departments are interdependent, as shown in Figure 2. When an organization has several interdependent subsystems, uncertainty about supply and demand might make it difficult for decision-makers to communicate with all the roles with which they are interdependent. The design solution for this is to create a mechanism that permits coordinated action across large numbers of interdependent roles and subsystems [1,19], and to transfer the decision points downward in the hierarchy to the points where the action takes place in order to ensure flexibility with increasing uncertainty.

One way to do this is to create lateral relationships (i.e., horizontally, outside the hierarchical level) to develop collaborative decision-making processes that cut across lines of authority and to increase the decision-making power of lower levels in the hierarchy. There are various design options, as discussed in Galbraith [1], from direct contact between managers who share a problem but have no formal decision power to a matrix with dual authority (e.g., technical and formal authority) in the organization. This aligns with Weick’s [17] model of organizing, in which decisions to identify appropriate responses to supply and demand uncertainty are made through collective interdependent communication.

In the example shown in Figure 2, the hospital can adopt the “integrating” role discussed in Galbraith [1]. At the operational level, an integrating role is created to acquire aggregated information (i.e., coarse-grained, as explained earlier) about the number of occupied beds in the ICU and the IWs. When a supply–demand mismatch occurs in a department, the role will have enough decision power to prioritize where patients should be admitted into wards. Having decision power at lower levels of the hierarchy reduces management’s effort and time, hence minimizing the effort needed for information processing to allocate patients to wards.

### 2.3. Entropy and Information Processing Structure

A given system design follows a specific structure in which information is gathered and processed. An information entropy model can be used to measure the amount of information [7]. Entropy is defined as a measure of randomness or disorder in a system [6,9]. One may ask why we should focus on entropy. It is because information entropy values can be assigned to specific structures so that we can compare them in order to gain insights into the factors that lead to effective decision-making. To calculate the information entropy (*H*), we can follow Shannon’s information entropy formula [8]. If the average uncertainty associated with an outcome is represented by discrete random variable *X*, Shannon’s information entropy of a discrete random variable of *X*, with *n* outcomes (*x*1, *x*2, …, *xn*), is as follows:

(1) $$H\;\left(x\right)=-\sum_{i=1}^{n}P\left(xi\right)\log_{2}P\left(xi\right)$$

Zhang and Xiao [7] defined the amount of information needed to describe the expected states of a system as structural entropy. As stated earlier, when a hospital system is characterized as having several workstations (i.e., departments) connected in a network, uncertainty about patients’ required positions in the system can cause delays in the decision-making process. We refer to this as positional uncertainty [6]. Furthermore, when departments have multi-skilled nurses, there is uncertainty about where to allocate nurses to the needed departments in the system, and this can also lead to delays in decision-making. We refer to this as task allocation uncertainty. Positional and task allocation uncertainty can both be measured with information entropy.

- Positional entropy

Given a specific system structure, we need the information of Pij, that is, the probability of a patient i (i=1, 2, …, N) progressing to department j (j=1, 2, …, M). For every i, $\sum_{j}P_{ij}=1.$ Having Pij, the positional entropy of a patient in department j can be calculated as follows:

(2) $$H_{j}=-\sum_{j=1}^{M}\left(Pij\log_{2}Pij\right)$$

The positional entropy of all patients in the system is the following:

(3) $$H_{s}=-\sum_{i=1}^{N}\sum_{j=1}^{M}\left(Pij\log_{2}Pij\right)$$

where $H_{s}$ is the positional entropy of the system, $P_{ij}$ is the probability of patient i (i=1, 2, …, N) progressing to department j (j=1, 2, …, M), N is the number of patients, and M is the number of possible states (i.e., departments) for patient i.

- Task allocation entropy

To measure task allocation entropy, we followed Shuiabi et al. [28] in conceptualizing entropy as a measure of flexibility. Shuiabi et al. [28] applied entropy to measure the relative demand for products, which is the ratio of time spent on processing a product to the total processing time for all products. When workstations can handle more products (i.e., they are flexible), the entropy associated with the process also increases. We applied the same concept to using multi-skilled nurses who can care for various patient groups: Pij, that is, the probability of a patient i (i=1, 2, …, N) being cared for by nurse type *j* (j=1, 2, …, M). For every i, $\sum_{j}P_{ij}=1.$ The relative demand *P* of patient *i* being cared for by nurse type *j* is as follows:

(4) $$P_{ij}=\frac{Ch}{\sum_{i=1}^{M}Ch}$$

The task allocation entropy of nurse type *j* is the following:

(5) $$H_{nj}=-\sum_{j=1}^{N}\left(Pij\log_{2}Pij\right)$$

The task allocation entropy of all nurses in the system is the following:

(6) $$H_{ns}=-\sum_{i=1}^{N}\sum_{j=1}^{M}\left(Pij\log_{2}Pij\right)$$

where $H_{ns}$ is the task allocation entropy of the system, N is the number of patient groups, M is the number of patient groups that nurse type *j* handles, and Ch is the hours caring for patient *i* by nurse type *j*.

- Extremum of information entropy

As stated above, probabilities are needed to calculate entropy, which depends on the available information in the system. When such information is unknown, assumptions are made to estimate probability. Instead of using an estimation, we can consider the extremum of information entropy [6].

The extremum of information entropy is used to discover the probability distribution, leading to the highest value for this uncertainty, thereby assuring that no information is carelessly assumed. That is, for the positional entropy example, we acknowledge that it is impossible to say that a patient is more likely to be in department j. When assuming the extremum of information entropy, the patient has an equal probability of being in any department j within the system.

- Multiplicity perspective and entropy

As stated earlier, information entropy values can be assigned to specific structures of information processing in order to compare them so that insights into the factors that lead to effective decision-making can be gained. In this respect, assigning the information entropy is the work that has to be performed to obtain information about situations. The multiplicity perspective can be used to evaluate the number of ways different outcomes can occur [29]. In this regard, the more independent options a department has, the more uncertainty it has in explaining a situation; hence, the higher the entropy will be.

## 3. Method

In this study, we applied information theory with entropy as the central concept to assess the effectiveness and costs of an organizational structure and its coordinated processes. To generate an in-depth understanding of the design strategy to enable the closed-loop system, we need to illustrate hospitals’ differentiation and integration dynamics. We applied the entropy analysis to the case setting of the perinatology care system at Radboud University Medical Centre (Radboudumc), Nijmegen, The Netherlands. Perinatology (also known as maternal–fetal medicine) is a rapidly growing field that is concerned with delivering care to mothers and newborns. A perinatology care system is often modeled after a complete hospital system, with pre- and post-care bed units and various levels of care units [30], though frequently on a smaller scale. Although the care in the neonatology department and the obstetrics department in Radboudumc is configured separately (i.e., departments are differentiated), the two departments are interdependent regarding patient demand; hence, integration mechanism is needed.

### 3.1. Case Study: Perinatology Care System, Radboud University Medical Centre

**Figure 4:**
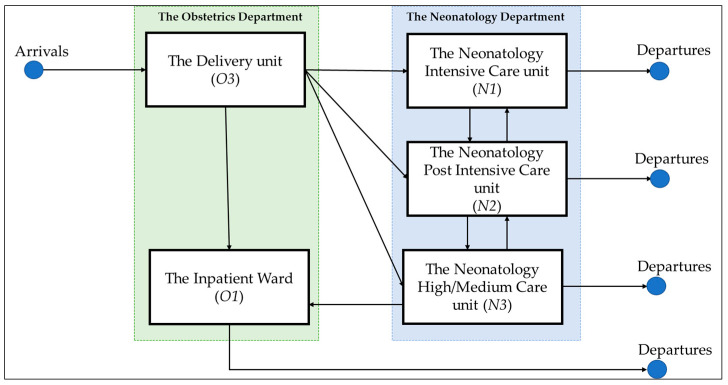
Newborn patient flow in perinatology care system.

The perinatology care system at Radboudumc focuses on newborn care pathways, as shown in Figure 4. In this example, when a baby is born in the obstetrics department (i.e., the delivery unit), the baby can proceed to four possible units: the neonatology intensive care unit (NICU), the high-care (HC), the medium-care unit (MC), or the inpatient wards of the obstetrics department (IW). The statistical probability (Pij) that a patient will progress to these departments is obtained from historical patient movements recorded in the hospital information system (HIS) in 2017. The skill matrix (i.e., the type of nurses who qualify to work in a specific department) was obtained through interviews with the head nurse of each department. We also obtained the number of nurses who can work in each unit.

### 3.2. Structural Entropy Scenarios

Using this case study, we explored six scenarios to find ways to minimize the structural entropy (positional and task allocation entropy), as shown in Table 1. The positional entropy is calculated with Formulas (2) and (3), while task allocation entropy is with Formulas (5) and (6), as presented in Section 2.3.

## 4. Results

**Figure 5:**
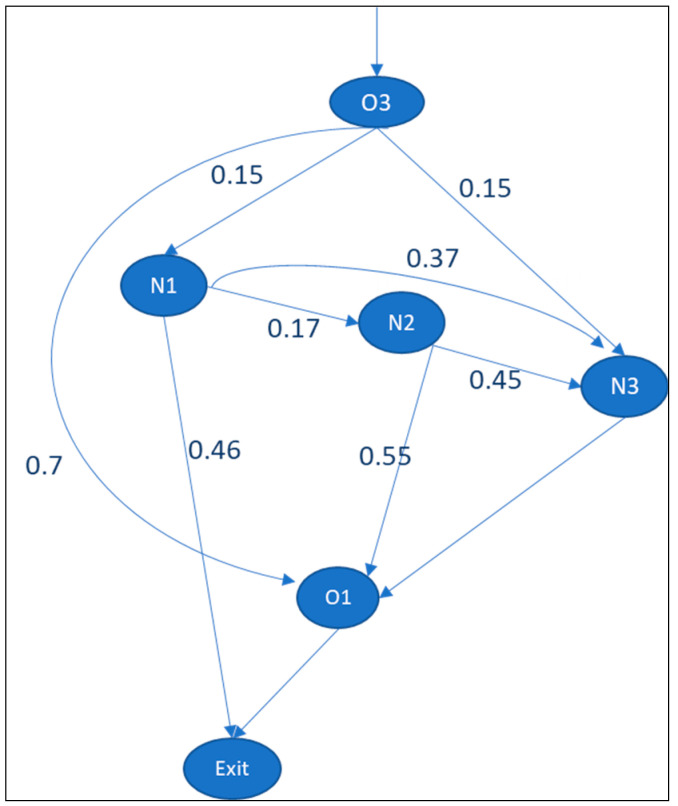
$P_{ij}$ values for babies born in delivery room of Radboudumc. Departments are delivery room (O3), nursery ward (O1) for healthy newborns, neonatal intensive care unit (N1) for newborns who need intensive treatment, post-intensive care unit (N2), and high- and medium-care unit (N3).

For the analysis, the first step is to create the information point structure. The department structure can be identical to the information point structure. This means that every department acquires and processes information for its decision-making process. The Pij values for newborns analyzed from the data extracted from the HIS are given in Figure 5.

### 4.1. Scenario 1: Baseline

Based on Formulas (2) and (3), Table 2 provides the positional entropy of a patient in department j and of all patients in the system. As shown in the table, the $H_{j}$ value for patients from N2 to O1 and N3 is 0.992 bits. The lower entropy of this path (N2 to O1 and N3) indicates that it exhibits less uncertainty than N1 to N2, N3, and exits ($H_{j}$ is 1.481 bits). The total positional entropy of the system is 3.65 bits (E1.a).

If we focus on the task allocation entropy of nurses in the departments, we can calculate the system’s entropy given the nurse matrix design, as shown in Table 3. The nurses in this scenario can only work in their dedicated departments. The care hours per patient per day are given in Table 4.

Analyzing the average occupied patient beds per day, there were 12 occupied beds per day at N1, 4 at N2, 7 at N3, 28 at O1, and 4 at O3. Table 5 provides the expected total care hours/patient/day shift. The task allocation entropy based on the nurse matrix is 1.86 bits (E1.b), as shown in Table 6.

### 4.2. Scenario 2: Extremum Condition

Based on the extremum information, Table 7 presents the system’s total positional entropy, which is 4.168 bits (E2.a).

As for the task allocation entropy, Table 8 presents the system’s entropy based on the extremum information entropy of expected patient demand in the departments, which is 2.23 bits (E2.b).

### 4.3. Scenario 3: Pooling Inpatient Wards

**Figure 6:**
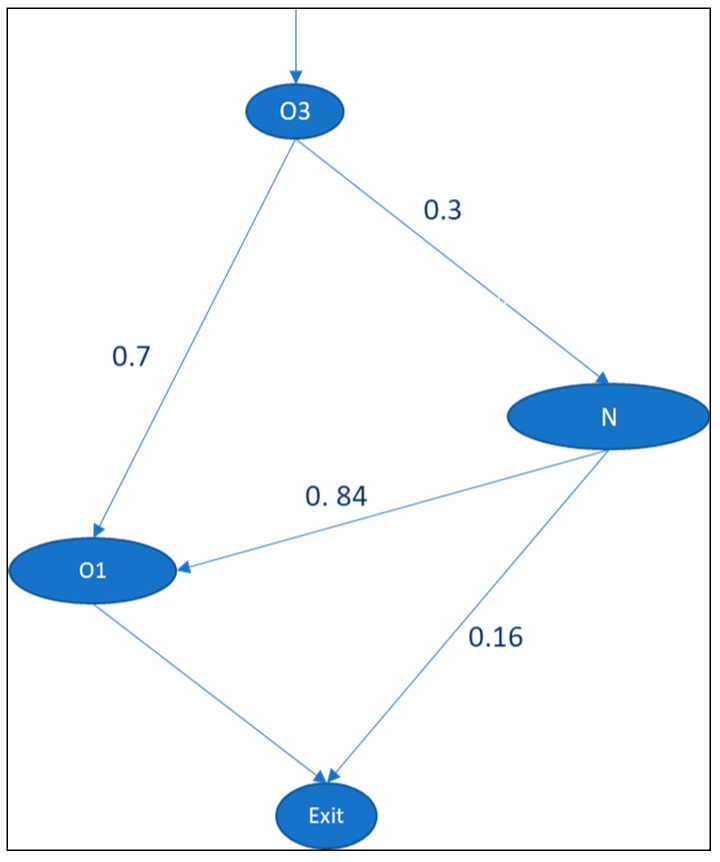
Scenario in which N1, N2, and N3 are pooled into N.

In this scenario, a buffer is created by pooling N1, N2, and N3 beds into the neonatology department. Hence, when babies are born in O3, they can proceed to either O1 or the neonatology department (N1, N2, or N3). In other words, N1, N2, N3 are physically pooled through a hierarchy. The new information point structure is given in Figure 6. The total positional entropy of the system, which is 1.51 bits (E3.a), is given in Table 9.

The strategy of pooling inpatient wards implies that nurses in N1, N2, and N3 are interchangeable, so the type of nurses is stated as N. In this scenario, the planner of N1, N2, and N3 is centralized. The analysis of average occupied patient beds per day shows that there were 23 occupied beds per day in the newly pooled N ward, 28 in O1, and 4 in O3. The new nurse matrix is given in Table 10. The task allocation entropy based on the nurse matrix is 1.41 bits (E3.b), as shown in Table 11.

#### Multiplication Analysis

Although the system (global) entropy (1.51 bits for positional entropy and 1.41 bits for task allocation entropy) is smaller than the baseline model (3.65 bits for positional entropy and 1.87 bits for task allocation entropy), the new (local) entropy of the pooled ward N is now higher than the baseline model.

- For the positional entropy, the local entropy of the pooled ward (0.521 bits; Table 9) is slightly higher than that of N1 (0.411 bits; Table 2) and N3 (0.411 bits; Table 2) in the baseline scenario.
- For the task allocation entropy, the local entropy is 0.52 bits (Table 11). The entropy of the baseline scenario is 0.52 bits for N1, 0.14 bits for N2, and 0.40 bits for N3.

This can be explained in terms of the multiplicity perspective: the more independent options a department has, the more uncertainty there is in explaining a situation. From the global perspective, the baseline model has several historical state options for where a patient can be in the system (O1, N1, and N3). When N1, N2, and N3 are pooled into the N ward, the system’s state options are now limited to either O1 or the newly pooled N ward. In the latter situation, the global decision freedom is reduced due to the decrease in the number of states, which reduces the associated global entropy. However, it increases the local decision freedom and associated local entropy in the pooled department because it covers more patients (i.e., there is more decision freedom) than in the previous situation.

Nevertheless, the gain of the pooled situation is that the central decision maker no longer needs to consider the situation of the pooled department at the micro level. From the perspective of the central decision-maker, all patients and nurses in the pooled department are the same. Handling the differentiation of patients and nurses is now a task that is delegated to the pooled department. This delegation reduces complexity, as the number of states to be considered decreases.

### 4.4. Scenario 4: Self-Contained Task

**Figure 7:**
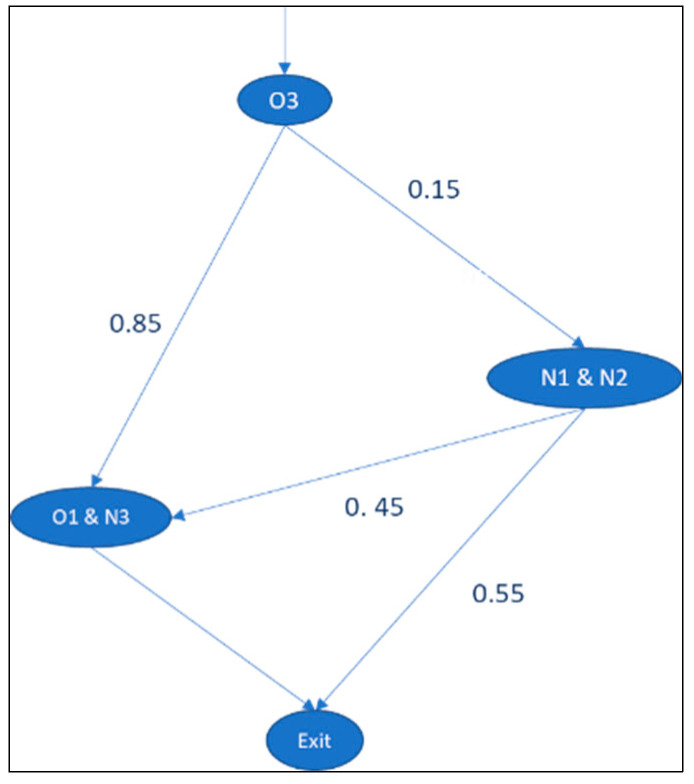
Scenario in which nurses from N3 and O1 are interchangeable.

In this design, the wards are not physically grouped; however, patients can be admitted to alternative wards when the primary ward is fully occupied. This is a reciprocal pairs cross-training policy [31], whereby units pair up, and each unit cross-trains one nurse to serve in the paired unit. In our study, nurses were grouped based on product classifications: intensive/post-intensive care and high/medium/normal care. The new information processing points based on the nurses’ structure are given in Figure 7. The total positional entropy of the system is 1.6 bits (E4.a).

In this strategy, nurses from N3 and O1 are interchangeable due to their skill requirements, with N1 and N2 being similar, as shown in the nurse matrix in Table 12. Here, planners are decentralized to each ward, as the wards are not physically pooled. The task allocation entropy based on the nurse matrix, which is 2.79 bits (E4.b), is presented in Table 13.

### 4.5. Scenario 5: Investment in Vertical Information System

**Figure 8:**
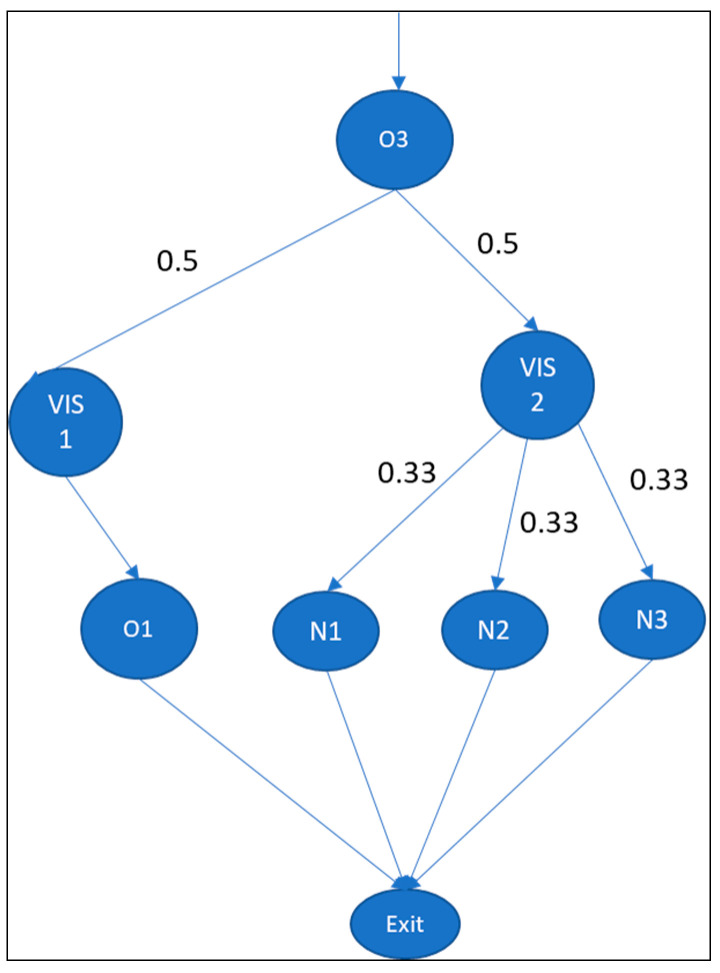
Scenario with vertical information system (VIS).

Investing in the vertical information system (VIS) increases the speed of information gathering and processing due to the formalization of language [1], such as by workload aggregation. In our case study, the hospital installed two VISs, each designed to acquire workload information from interdependent (local) departments. As shown in Figure 8, we assumed an extremum of information entropy; that is, a patient has an equal probability of being in any state within the system. The total positional entropy of the system is 1.78 bits (E5.a).

As for the allocation of nurses, the task coordination remains local in the departments, hence this scenario is similar to scenario 2. The task allocation entropy based on the nurse matrix is 2.23 bits (E5.b).

### 4.6. Scenario 6: Creation of Lateral Relations

**Figure 9:**
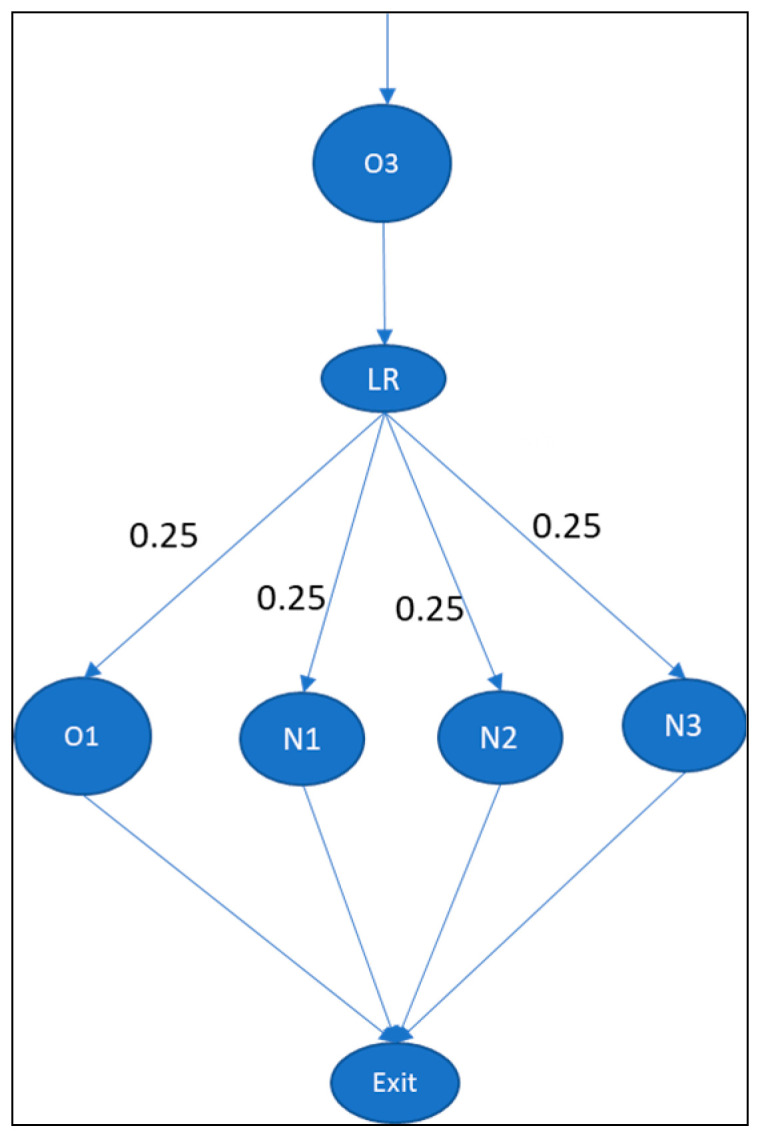
Scenario with lateral relation (LR).

The creation of lateral relations is developed to enable collaborative decision processes that cut across department lines (e.g., O1, N1, N2, and N3). The hospital adopts the “integrating” role as discussed in Galbraith [1], by which the decision power is transferred downward in the hierarchy to the points where the action takes place in order to ensure flexibility with increased uncertainty. With this investment, the processing points of the hospital are changed to position the lateral relation in the hospital system, as shown in Figure 9. The role acquires information about the number of occupied beds in departments. We assume an extremum of information entropy; a patient has an equal probability of being in any state within the system. The total positional entropy of the system is 2 bits (E6.a).

As for the allocation of nurses, the task coordination remains local to the departments, hence this scenario is similar to Scenario 2. The task allocation entropy based on the nurse matrix is 2.23 bits (E6.b).

## 5. Discussion

- **What is the optimal care system configuration (i.e., how are departments partitioned) to minimize uncertainty?**

**Figure 10:**
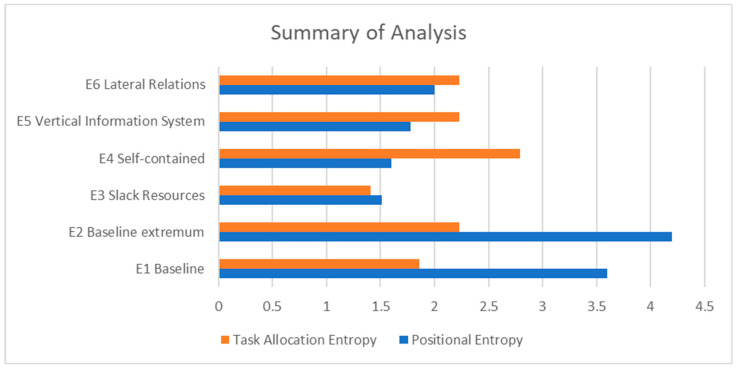
Summary of analysis.

Summaries of six scenarios for the perinatology care system are given in Table 14 and Figure 10. For the studied system, the optimal care design to minimize uncertainty is shown by scenario 3: creating slack resources by pooling wards. The pooling of wards is achieved by merging the nurses’ capacity and the beds. Our results agree with previous studies about pooling systems together (e.g., combining different types of patient groups and their capacity), providing the possibility to reduce the average number of capacity buffers needed in the system [25,26]. Particularly, we agree with the results of Gong et al. [6], showing that fewer departments in a system will result in lower structural entropy, as less time is required to search for efficient solutions. The lower entropy further indicates less decision-making time, thus reducing patient flow delays.

When physical pooling is not possible, hospitals can create self-containment tasks (E4) through which nurses are cross-trained between several selected wards. This way patients can be admitted to these wards when the primary ward is fully occupied. Although we agree with Inman et al. [31] that cross-training can benefit a system with a highly uncertain environment, our analysis of the studied system shows that the associated task allocation uncertainty is higher than in the baseline scenario. In scenario 4, the search space for solutions is larger than in the baseline scenario because nurses are still decentralized in each ward, as the wards are not physically pooled. This implies that implementing a cross-training policy could increase the complexity of allocating nurses to the wards.

Alternatively, the studied system can invest in a vertical information system (VIS) (scenario E5) or lateral relations (scenario E6) to increase information processing capability. In scenario 5 (investment in VIS), the information of all wards is aggregated only to report their workload (i.e., the number of occupied beds), and not details on patient mismatches. The lower entropy provided in scenario 5 compared to the baseline scenario indicates less decision-making time, thus reducing patient flow delays. The situation in scenario 5 is similar to aggregation by workload with the CONWIP system, as discussed by Gong et al. [6]. CONWIP is a successful method of dealing with uncertain environments studied by previous researchers [23,27]. However, with a CONWIP system, the organization deliberately accepts that variations from the required workload are possible. As Pettersen and Segerstedt [27] observed, the coefficient of the variation of lead time increases when the workload increases, which can further disrupt the patient flow. To overcome this, organizations can build slack resources explicitly to deal with the variation, as stated earlier.

For lateral relations (scenario E6) at the operational level, an integrating role is created to acquire aggregated workload information about the number of occupied beds in the ICU and IWs, as well as to make decisions regarding eventual mismatches. This is similar to what was discussed by Weick [17]; that is, organizations can best resolve a highly uncertain environment through collective interdependent communication and information processing activities that help reduce uncertainty and identify appropriate responses. The lower entropy of scenario 6 compared to the baseline scenario indicates that information about patients’ positions in the system is acquired and processed efficiently by the lateral role.

- **How does the distribution of authority minimize uncertainty?**

According to Weick’s [17] model of organizing, one option to deal with uncertainty is to manage the distribution of authority within the organization. In this study, we evaluated where the decision authority of allocating patients and nurses to wards should reside. When the decision authority to allocate patients and nurses to wards is centralized in pooled wards by a global planner (scenario 3), the planner no longer needs to consider the situation of the pooled department at the micro level. All patients and nurses in the pooled department are considered the same, which decreases the search space of allocation. Thus, scenario 3 minimizes the delay needed to process information regarding where to allocate patients and nurses compared to the baseline scenario (E1). As for scenario 4, the planners are still decentralized in each ward. As the amount of search space (i.e., nurses’ skills) increases because of the cross-training arrangements, so does the complexity of allocating them compared to the baseline model (E1).

As for scenario 5, aggregated feedback from the operational level is cascaded up to the tactical level, where there is a global perspective; therefore, managers can make appropriate decisions about out-of-scope mismatches. Compared to the baseline model, assuming maximum entropy (E2) due to the aggregated information (e.g., workload as the number of occupied beds), the output diversity and thus the effort needed for information processing to allocate patients to wards are eventually reduced. In scenario 6, the decision power to solve out-of-scope mismatches is transferred downward in the hierarchy, at the operational level, to the points where the action takes place. Compared to the baseline model and assuming maximum entropy (E2), in scenario 6, uncertainty decreases as a certain amount of information processing regarding the expected positions of patients remains local, reducing decision delay.

- **How can the entropy concept be used to evaluate hospital organization and information processing?**

As discussed previously by Zhang and Xiao [7] and Gong et al. [6], entropy analysis can be used to measure the uncertainty related to the work that has to be performed to obtain information about certain events. Particularly, two types of entropy appear to be important: positional entropy and task allocation entropy. These are different dependent variables on the organizational design scenarios. However, entropy itself is not an absolute method for determining optimal organizational structures and coordination processes. The interpretation is subjective in terms of how the uncertainty associated with the environment is perceived.

When the environment is highly uncertain, the system needs a high degree of decision freedom to mitigate the situation when the actual output deviates from the desired output caused by uncertainty [17,23]. A high degree of decision freedom implies many state spaces, resulting in high entropy [28]. Hence, for organizations with a highly uncertain environment, an organizational design with high entropy structural solutions is desirable in that it can reflect the organization’s operational flexibility in dealing with an uncertain environment. In the current case study, however, the task uncertainty belonging to the environment is relatively known, as indicated by the gathered historical data. Reflecting on this, minimizing the structural entropy is desirable to optimize hospital operations.

## 6. Conclusions

As stated earlier, a hospital needs to be treated as a closed-loop system characterized by a high degree of integration between departments to deal with uncertainty [5,21]. In this study, we applied information theory using the entropy concept to assess the effectiveness of a hospital’s organizational structure and coordinated processes.

### 6.1. Practical Implications

As was suggested, the choice of an integration and coordination strategy depends on the characteristics of the tasks (uncertainty) in the organization’s segments [19]. In this study, it was our choice to use a case study with relatively known data (i.e., environmental), as our aim was to minimize the structural entropy. The aim could be different for other environmental characteristics. In view of this, our analysis shows that entropy itself is not an absolute method to determine optimal organizational structures and coordination processes. The interpretation is subjective in terms of how the uncertainty associated with the environment is perceived. Still, entropy can be used as a concrete way of measuring the effectiveness of an organizational design. Furthermore, when partitioning or structuring an organization, it is essential to quantify the search space for solutions. From the multiplicity perspective, the more independent search spaces there are in a department, the more uncertainty there is in explaining the situation. In our case settings, we showed that pooling the nurses’ capacity and beds (scenario E3) provided the optimal configuration for minimizing positional and task allocation uncertainty, as the number of states to be considered was decreased compared to the baseline scenario.

Pooling departments can minimize uncertainty, although it is often not a simple decision. Resource capacity is grouped into departments based on the specialization under consideration. When departments focus on a selected range of care products, they are better able to control the fulfillment of tasks for achieving higher quality and having more control over costs [23]. One way to exploit the benefit of pooling is to create (nurse) flexibility within the existing capacity to deal with uncertainty and variability in the demand for patient car [23,26,32] In the current study, we portrayed this as the creation of a self-contained task (scenario 4, in which nurses can work in more than one department), and we also showed that positional entropy is minimized under the self-contained task scenario, but not task allocation uncertainty. Given this, hospitals must accept the consequences of extra coordination work (and time) if nurses are expected to work in more than one department.

Additionally, not all nurses are willing to be reallocated to other wards (scenario 4). At the same time, not all nurses are required to be flexible. The effectiveness of flexibility is limited to a specific bandwidth [26]. Our previous study [26] showed that exercising flexibility below or above a specific bandwidth will not substantially improve the system’s performance. Further research is needed to evaluate when and where other types of redundancy are needed for the effectiveness of a closed-loop system.

### 6.2. Limitations and Future Research

The current study is not without limitations. First of all, the degree of language formalization and aggregation plays a role in minimizing and responding to uncertainty [1]. When the language of feedback information is formalized and aggregated through coarse graining, hospitals can reduce output diversity and the effort required for information processing. In this study, the vertical information system (scenario E5) and lateral relation (scenario E6) deploy a CONWIP system, in which the information is abstracted to the workload level (i.e., occupied beds). Other coarse-graining methods in information processing organizations are an interesting topic for further research.

In this study, we used entropy as the central concept to assess the effectiveness and costs of the organizational structure and its coordination processes. Here, the cost is defined as the time needed for information assessment and processing, and the time depends on the efforts to gather and share information with others. Minimizing entropy implies that the organization exerts less effort on information processing and its coordination processes, thus minimizing the time needed for information assessment and processing. Although further research is needed to empirically quantify the costs, we already provide some insights into which configurations and integration mechanisms are effective against uncertainty.

In addition to the two structural entropies mentioned in this article, hospital departments can also apply operational entropy (such as in-control or out-of-control states) measures to evaluate the optimal scheduling horizon of a system. As Zhang [7] discussed, the degree of deviation due to uncertainties can be controlled by setting up a reasonable schedule horizon. With the appropriate schedule horizon in hospital departments, the need for nurses’ rescheduling will be at a minimum, hence the efforts to process the needed information. Empirical research on this is necessary to increase the understanding of other entropy applications in hospital operations.

During the decision-making process of hospital operations, Bayes theorem and maximum entropy can be used to determine a probability distribution of an event based on certain information. While Bayes decision-making is based on information gain (i.e., updating what we think of an event based on prior knowledge), maximum entropy decision-making is based on a priori with limited or no prior knowledge of the current event. For the context studied in this article, the decision-making process based on the maximum entropy is chosen because the planner admits patients based on an aggregate diagnosis (i.e., a priori), although she/he expects that, during the hospitalization, the information about the patients increases. Hence, the insight about the nurse workload also improves. Further, one may ask “who should decide on what information?”. To deal with (environment) uncertainty, the department manager, the nurses, or both should have certain decision autonomy. The specific answer to this is related to the organizational design choices of the hospital. Van der Ham et al. [20] emphasized that there is a relation between the degree of differentiation of the organization structure and the degree of professional autonomy (i.e., central versus decentral planning and control). In our study, we argue that a central planner can make the decision to reallocate nurses centrally, with a coarse-graining information processing and governed by central rules. Because the planner no longer needs to consider the situation of the department at the micro level, the time needed to process information regarding where to allocate patients and nurses is minimized. Although a central planning and control system may be necessary to plan patients’ processes and the required capacity in a closed-loop system, van Merode et. al. [33] highlighted the importance of knowledge management for decentral departments with highly uncertain environments. Through knowledge management, departments exercise systemized knowledge sharing between nurses to improve the knowledge (i.e., information) about the patients’ conditions, eventually leading to more responsive care delivery. On this note, given the recent review of nurse professional autonomy and their ability to influence working practices [34], further research is needed to investigate the role of nurses in minimizing delays during decision-making.

## Figures and Tables

**Table 1:** Perinatology care system scenarios.

Scenario	Independent Variable	Dependent Variable 1: Positional Entropy $H_{s}$	Dependent Variable 2: Task Allocation Entropy $H_{ns}$
1	Baseline	E1.a	E1.b
2	Baseline maximum entropy	E2.a	E2.b
3	Slack resources (i.e., pooling inpatient wards through hierarchy)	E3.a	E3.b
4	Self-contained task (i.e., multi-skilled nurses for inpatient wards)	E4.a	E4.b
5	Vertical information system	E5.a	E5.b
6	Lateral relations	E6.a	E6.b

**Table 2:** Positional entropy results of scenario 1 in perinatology care system.

From/To	O3	O1	N1	N2	N3	Exits	$H_{j}$
Arrival	0						0
O3		0.36	0.411		0.411		1.182
O1						0	0
N1				0.435	0.531	0.515	1.481
N2		0.474			0.518		0.992
N3		0					0

**Table 3:** Nurse matrix.

From/To	N1	N2	N3	O1	O3
N1	1	0	0	0	0
N2	0	1	0	0	0
N3	0	0	1	0	0
O1	0	0	0	1	0
O3	0	0	0	0	1

**Table 4:** Care hours/patient/day shift.

From/To	N1	N2	N3	O1	O3
N1	6.5	0	0	0	0
N2	0	4.5	0	0	0
N3	0	0	4	0	0
O1	0	0	0	2	0
O3	0	0	0	0	4

**Table 5:** Observed total care hours/patient/day shift.

Nurse Type	N1	N2	N3	O1	O3
N1	78	0	0	0	0
N2	0	18	0	0	0
N3	0	0	28	0	0
O1	0	0	0	56	0
O3	0	0	0	0	16

**Table 6:** Task allocation entropy results of scenario 1 in perinatology care system.

Nurse Type	N1	N2	N3	O1	O3	$H_{nj}$
N1	0.52					0.52
N2		0.14				0.14
N3			0.40			0.40
O1				0.52		0.52
O3					0.29	0.29

**Table 7:** Positional entropy results of scenario 2 in perinatology care system.

From/To	O3	O1	N1	N2	N3	Exits	$H_{j}$
Arrival	0						0
O3		0.528	0.528		0.528		1.584
O1						0	0
N1				0.528	0.528	0.528	1.584
N2		0.5			0.5		1
N3		0					0

**Table 8:** Task allocation entropy results of scenario 2 in perinatology care system.

Nurse Type	N1	N2	N3	O1	O3	$H_{nj}$
N1	0.52					0.52
N2		0.47				0.47
N3			0.46			0.46
O1				0.32		0.32
O3					0.46	0.46

**Table 9:** Positional entropy results of scenario 3 in perinatology care system.

From/To	O3	O1	N	Exits	$H_{j}$
Arrival	0				0
O3		0.36	0.521		0.881
O1				0	0
N		0.211		0.423	0.634

**Table 10:** Nurse matrix for scenario 3.

From/To	N	O1	O3
N	1	0	0
O1	0	1	0
O3	0	0	1

**Table 11:** Task allocation entropy results of scenario 3 in perinatology care system.

Nurse Type	N	O1	O3	$H_{nj}$
N	0.52			0.52
O1		0.53		0.53
O3			0.37	0.37

**Table 12:** Nurse matrix for scenario 4.

From/To	N1	N2	N3	O1	O3
N1	1	1	0	0	0
N2	1	1	0	0	0
N3	0	0	1	1	0
O1	0	0	1	1	0
O3	0	0	0	0	1

**Table 13:** Task allocation entropy results of scenario 4 in perinatology care system.

Nurse Type	N1	N2	N3	O1	O3	$H_{nj}$
N1	0.46	0.38				0.84
N2	0.24	0.19				0.43
N3			0.27	0.15		0.42
O1			0.51	0.40		0.91
O3					0.19	0.19

**Table 14:** Summary of positional entropy for numerical example.

Independent Variable		Dependent Variable	Positional Entropy	Task Allocation Entropy
Baseline		E1	3.6 bits	1.86 bits
Baseline maximum entropy		E2	4.2 bits	2.23 bits
Reduce need for information processing	Creation of slack resources	E3	1.51 bits	1.41 bits
	Creation of self-contained tasks	E4	1.6 bits	2.79 bits
Increase capacity for information processing	Investment in vertical information system	E5	1.78 bits	2.23 bits
	Creation of lateral relations	E6	2 bits	2.23 bits

## Data Availability

Not applicable.

## References

[1] Galbraith J.R. (1974). Organization Design: An Information Processing View. Interfaces.

[2] Radner R. (1993). The Organization of Decentralized Information Processing. Econometrica.

[3] Van Merode G.G., Groothuis S. (2003). Hospitals as complexes of queuing systems. Health Sciences Simulation.

[4] Kolker A. (2013). Interdependency of hospital departments, hospital-wide patient flows. Patient Flow: Reducing Delay in Healthcare Delivery.

[5] Munavalli J.R., Boersma H.J., Rao S.V., van Merode G.G. (2021). Real-time capacity management and patient flow optimization in hospitals using AI methods. Artificial Intelligence and Data Mining in Healthcare.

[6] Gong Q., Yang Y., Wang S. (2014). Information and decision-making delays in MRP, KANBAN, and CONWIP. Int. J. Prod. Econ..

[7] Zhang Z., Xiao R. (2009). Empirical Study on Entropy Models of Cellular Manufacturing System. Prog. Nat. Sci..

[8] Shannon C.E. (1948). A mathematical theory of communication. Bell Syst. Tech. J..

[9] Chappell D., Dewey G. (2014). Defining the Entropy of Hierarchical Organizations. Complex. Gov. Netw..

[10] Battini D., Persona A., Allesina S. (2007). Towards a use of network analysis: Quantifying the complexity of Supply Chain Networks. Int. J. Electron. Cust. Relatsh. Manag..

[11] Tushman M.L., Nadler D.A. (1978). Information Processing as an Integrating Concept in Organizational Design. Acad. Manag. Rev..

[12] Yu W., Zhao G., Liu Q., Song Y. (2021). Role of big data analytics capability in developing integrated hospital supply chains and operational flexibility: An organizational information processing theory perspective. Technol. Forecast. Soc. Chang..

[13] Huang P.Y., Pan S.L., Ouyang T.H. (2014). Information processing capability for operational agility: Implications from a Chinese manufacturer. Eur. J. Inf. Syst..

[14] Srivastava S., Singh R.K. (2020). Exploring integrated supply chain performance in healthcare: A service provider perspective. Benchmarking Int. J..

[15] Andersen A.R., Plesner A.L. (2022). Optimization of the organizational structure in hospitals to account for patients with multiple diseases. Artif. Intell. Med..

[16] Perryman-Starkey M., Rivers P.A., Munchus G. (1999). The Effects of Organizational Structure on Hospital Performance. Health Serv. Manag. Res..

[17] Weick K.E. (2012). Impermanent systems and medical errors: Variety mitigates adversity. Making Sense of the Organization Volume 2: The Impermanent Organization.

[18] Tippong D., Petrovic S., Akbari V. (2022). A review of applications of operational research in healthcare coordination in disaster management. Eur. J. Oper. Res..

[19] Lawrence P.R., Lorsch J.W. (1967). Differentiation and Integration in Complex Organizations. Adm. Sci. Q..

[20] Van der Ham A., Van Raak A., Ruwaard D., Van Merode F. (2022). Exploring changes in integration, differentiation, rules, coordination and performance following the introduction of a hospital planning centre: A case study. J. Health Organ. Manag..

[21] Munavalli J.R., Rao S.V., Srinivasan A., Van Merode G. (2019). Integral patient scheduling in outpatient clinics under demand uncertainty to minimize patient waiting times. Health Inform. J..

[22] O’Brien-Pallas L., Meyer R.M., Hayes L.J., Wang S. (2010). The Patient Care Delivery Model—An open system framework: Conceptualisation, literature review and analytical strategy. J. Clin. Nurs..

[23] Hopp W.J., Lovejoy W.S. (2014). Hospital Operations: Principles of High Efficiency Health Care.

[24] Bekker R., Roubos D. (2016). Flexible bed allocations for hospital wards. Health Care Manag. Sci..

[25] Litvak E. (2005). Optimizing patient flow by managing its variability. Front Office to Front Line: Essentials Issues for Health Care Leaders.

[26] Winasti W., Elkhuizen S., van Merode F., Berden H. (2022). Creating Coherence-Based Nurse Planning in the Perinatology Care System. Healthcare.

[27] Pettersen J.A., Segerstedt A. (2009). Restricted work-in-process: A study of differences between Kanban and CONWIP. Int. J. Prod. Econ..

[28] Shuiabi E., Thomson V., Bhuiyan N. (2005). Entropy as a measure of operational flexibility. Eur. J. Oper. Res..

[29] Dill K.A., Bromberg S. (2011). Molecular Driving Forces: Statistical Thermodynamics in Biology, Chemistry, Physics and Nanoscience.

[30] Cochran J.K., Bharti A. (2006). Stochastic Bed Balancing of An Obstetrics Hospital. Health Care Manag. Sci..

[31] Inman R.R., Blumenfeld D.E., Ko A. (2005). Cross-Training Hospital Nurses to Reduce Staffing Costs. Healthc. Manag. Rev..

[32] Green L.V. (2004). Capacity planning and management in hospitals. Operations Research and Health Care.

[33] Van Merode G.G., Groothuis S., Hasman A. (2004). Enterprise Resource Planning for Hospitals. Int. J. Med. Inform..

[34] Pursio K., Kankkunen P., Sanner-Stiehr E., Kvist T. (2021). Professional autonomy in nursing: An integrative review. J. Nurs. Manag..

